# Competitive Interaction of Phosphate with Selected Toxic Metals Ions in the Adsorption from Effluent of Sewage Sludge by Iron/Alginate Beads

**DOI:** 10.3390/molecules25173962

**Published:** 2020-08-31

**Authors:** Hanna Siwek, Krzysztof Pawelec

**Affiliations:** Department of Bioengineering, West Pomeranian University of Technology Szczecin, ul. J.Słowackiego 17, 71-434 Szczecin, Poland; krzysztof.pawelec@zut.edu.pl

**Keywords:** phosphorus removal, toxic metals, adsorption, alginate beads, sewage sludge

## Abstract

Wastewater is characterized by a high content of phosphate and toxic metals. Many studies have confirmed the sorption affinity of alginate adsorbents for these ions. In this study, the adsorption of phosphate from effluent of sewage sludge on biodegradable alginate matrices cross-linked with Fe^3+^ ions (Fe_Alg) was investigated. Kinetics and adsorption isotherms were tested in laboratory conditions in deionized water (DW_P) and in the effluent (SW_P), and in the same solutions enriched in toxic metals ions—Cu^2+^, Cd^2+^, Pb^2+^, and Zn^2+^ (DW_PM and SW_PM). Batch experiments were performed by changing the concentration of phosphate at constant metal concentration. Kinetics experiments indicated that the pseudo-second-order model displayed the best correlation with adsorption kinetics data for both metals and phosphate. The Freundlich equation provided the best fit with the experimental results of phosphate adsorption from DW_P and DW_PM, while the adsorption from SD_P and SD_PM was better described by the Langmuir equation. For tested systems, the affinity of the Fe_Alg for metal ions was in the following decreasing order: Pb^2+^ > Cu^2+^ > Cd^2+^ > Zn^2+^ in DW_PM, and Pb^2+^ > Cu^2+^ > Cd^2+^ > Zn^2+^ in SW_PM. The metals’ enrichment of the DW_P solution increased the affinity of Fe_Alg beads relating to phosphate, while the addition of the metals of the SW_P solution decreased this affinity.

## 1. Introduction

The growing world population causes intensification of food production, which generates an increasing use of fertilizers, including phosphate ones. The main industrial source of phosphorus (P) is phosphate, the resources of which, like oil and natural gas, are non-renewable. The results show that 70% of the global production of phosphate rock is currently produced from reserves which will be depleted within 100 years and combining this with increasing demand will result in a significant global production deficit, which by 2070 will be larger than the current production [1,2]. Phosphorus does not disappear like hydrocarbons after “consumption” but can be recycled. In accordance with the principles of sustainable development, attempts are made to search for alternative sources of phosphorus in the human food chain, e.g., in municipal waste. Municipal wastewaters may contain from 6 to 8 mg/L of total phosphorous [3,4]. The national P budgets in Central Europe show that municipal wastewater contains a P load that could theoretically replace 40% to 50% of the annually applied mineral P fertilizer in agriculture [5].

A particularly numerous group of methods for the recovery of phosphorus from wastewater and manure is the crystallization of struvite [6] or combustion [6,7,8]. An alternative method of phosphorus removal at various stages of wastewater and sewage sludge treatment can be adsorption of mineral phosphorus forms from the liquid phase. To this end, adsorbents containing iron compounds, mainly oxides and carbonates, e.g., allophane [9], neutralized red sludge containing 40–45% Fe_2_O_3_ [10], fly ash granulated and sintered at 1000–1350 °C [11] or goethite [12,13] were tested. Due to the high impact of pH and redox potential on the phosphorus binding process with the participation of minerals and iron-containing waste, these adsorbents have little application significance [14,15]. The second group of sorbents are adsorbents activated with multivalent metals, e.g., lanthanum-modified bentonite [16] or iron-modified: refined aspen wood fiber [17,18], coir pith [19], eggshell [20] and *Staphylococus xylosus* biomass [21]. All the adsorbents described above were tested only on a small scale, and their main shortage is difficult recovery after the phosphorus binding process. The third group of sorbents are hydrogel composites obtained on the basis of natural polysaccharides, e.g., alginates, the effective action of which has been repeatedly confirmed during purification of the aqueous environment from pigments, heavy metals and antibiotics. Alginate is a natural anionic polysaccharide extracted from algae, which is non-toxic and biodegradable. The major component of alginate is Na-salt of alginic acid with abundant free hydroxyl and carboxyl groups distributed along the backbone chain of the polymer. It can pass through an irreversible chemical process with polyvalent cations (except magnesium) to form a crosslinking bond, and finally forming the thermo-irreversible gel [22]. Alginate cross-linked with multivalent metals, e.g., calcium, forms structures with a large specific surface [23,24]. After chemical modification, it can be converted into adsorbents with anionic sorption capacity. By such modification using Fe(III) or Zr(IV) compounds, adsorbents for purifying water from oxygen anions containing Se(IV), Cr(VI) and As(V) [25,26] or P [27,28] compounds were obtained.

Wastewater is characterized by a high content of toxic metals, examples of metal contents in municipal wastewater are: 0.75 mgCu^2+^/L, 1.13 mgZn^2+^/L and 0.4 mgPb^2+^/L, whereas in industrial wastewater: 2.13 mgCu^2+^/L, 17.3 mgZn^2+^/L, 6.1 mgPb/L and 0.1 mgCd^2+^/L [29]. Many studies have confirmed the sorption affinity of alginate adsorbents for these metals [22] so the metals can compete in the phosphate adsorption process. The aim of this paper was to study the adsorption of phosphate in a multicomponent system on biodegradable alginate matrices cross-linked with Fe^3+^ ions (Fe_Alg). The tests were carried out in effluent water generated during the processing of sewage sludge from sewage treatment plants, and the tested effluent has been enriched with selected multivalent metals. To assess the competitiveness of effluent components for phosphate adsorption, analogous comparative studies were carried out in ion solutions prepared in deionized water.

## 2. Results

### 2.1. Phosphate Adsorption Kinetics

**Figure 1:**
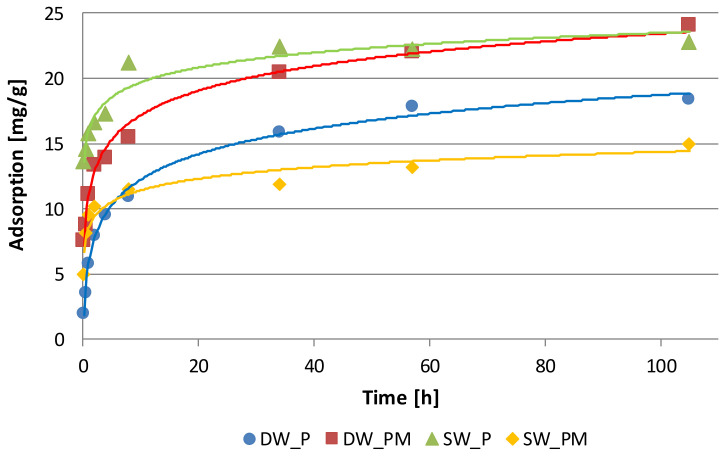
Adsorption kinetics of phosphate ions by alginate matrices cross-linked with Fe^3+^ ions. DW_P—deionized water enriched with phosphate, SW_P—effluent from a sludge enriched with phosphate, DW_PM—deionized water enriched with phosphate and metal ions, SW_PM—effluent from a sludge enriched with phosphate and metal ions.

The kinetics of phosphate binding from solutions without and with addition of metal ions by Fe_Alg matrices showed that the amount of adsorbed phosphate increased over time (Figure 1). The main goal of the kinetic studies was to determine the time after which the tested systems reach the state of adsorption equilibrium, and thus how long the adsorption time will be in the study of adsorption isotherms.

In the examined systems, the equilibrium was established after 56 h, after which 70.1% and 59.2% phosphate were removed from the DW_P and DW_PM solutions and 76.5% and 63.3% from SW_P and SW_PM, respectively. It has to be noted that that from 53.54% (for SD_PM solution) to 92.83% (for SW_P solution) of the maximum sorption capacity is achieved within 8 h.

Kinetics experiments indicated that the pseudo-second-order model displayed the best correlation with phosphate adsorption kinetics data for all the tested systems. The adjusted determination coefficients for the linear *t*/*a_t_* dependence as a function of time ranged from 0.996 to 0.999 (Table 1). The presence of metals in the DW_PM solution increased values of *a_eq_* and *V_o_*, while in the SW_PM solution, these values decreased. Values of adsorption phosphate at equilibrium state are consistent with the adsorption capacities obtained from the phosphate adsorption isotherms presented in this study. The values of *k*_2_ calculated for the models describing adsorption phosphate from SW_P and SW_PM solutions were higher compared to *k*_2_ values calculated for DW_P and DW_PM solutions.

**Figure 2:**
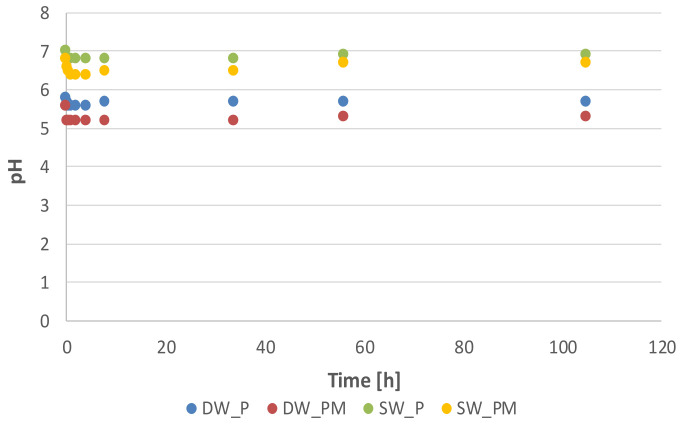
Changes in the pH of the tested systems during the adsorption process. DW_P—deionized water enriched with phosphate, SW_P—effluent from a sludge enriched with phosphate. DW_PM—deionized water enriched with phosphate and metal ions, SW_PM—effluent from a sludge enriched with phosphate and metal ions.

As pH is an important factor affecting the removal of ions from aqueous solution, the pH changes of the solutions during adsorption were studied. In the conducted experiment, the pH of effluent water was higher in comparison with deionized water, i.e., 7.0 and 5.8, respectively. During the process, the pH changed to a small extent, while the most significant changes were found for the DW_PM solution, in which the pH decreased by 0.6 (Figure 2). A greater decrease of the pH in DW_PM and SW_PM solutions compared to DW_P and SW_P indicates a competition for binding sites’ metal ions and remaining protons on the alginate matrix.

**Figure 3:**
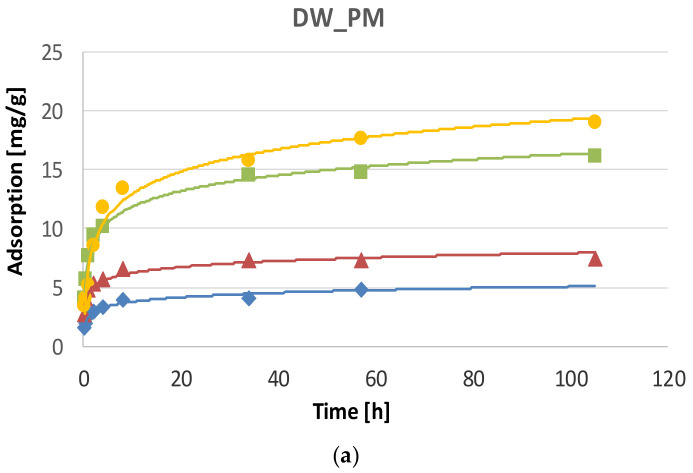

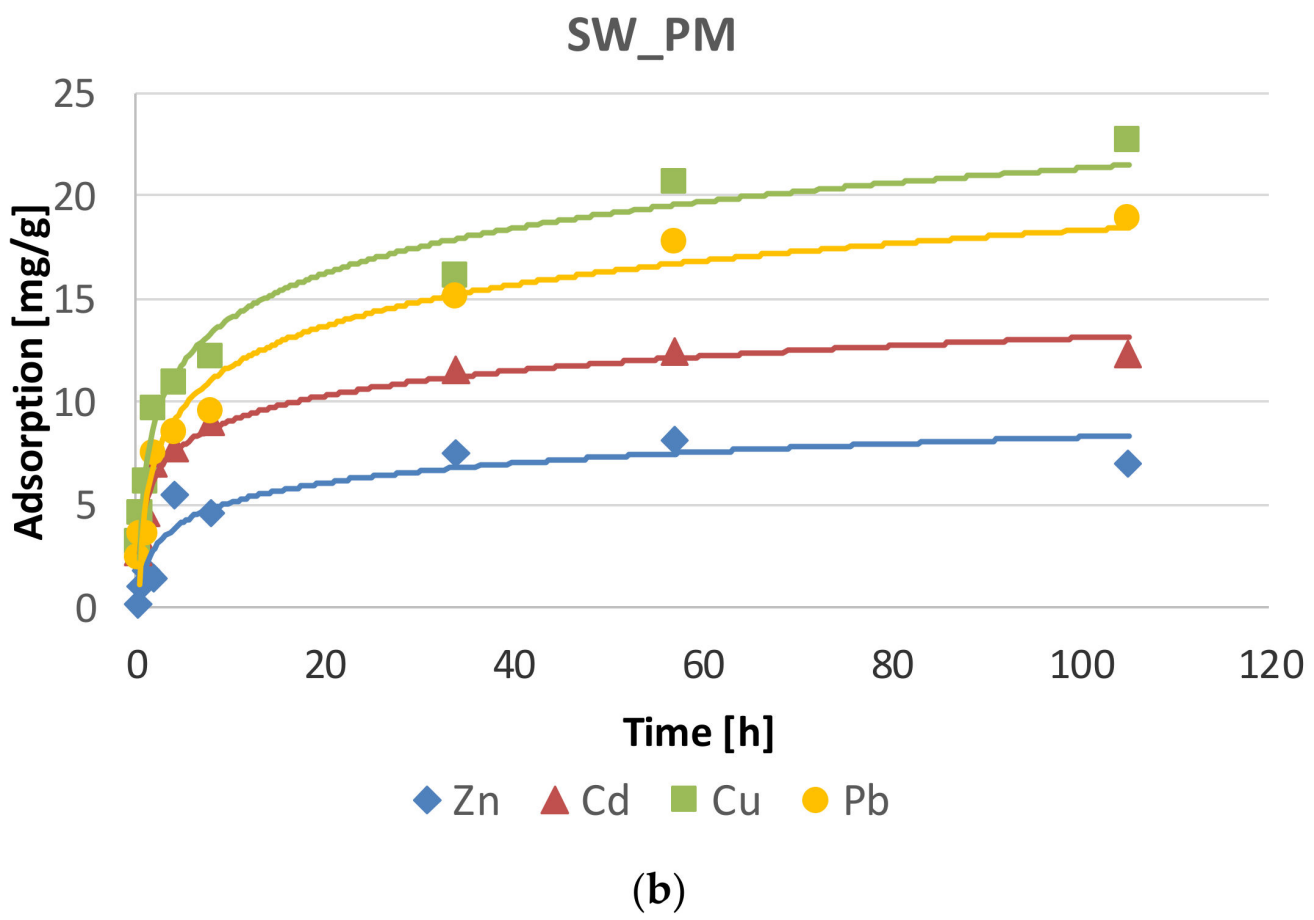
The kinetics of metal ion binding in the presence of phosphate by alginate matrices cross-linked with Fe^3+^ ions in: (**a**) deionized water enriched with phosphate and metal ions (DW_PM) and (**b**) effluent from a sludge enriched with phosphate and metal ions (SW_PM).

Changes in the content of investigated metals in alginate capsules during the phosphate adsorption process showed that the most lead was removed from DW_PM, whereas the most copper from SW_PM solutions (Figure 3). The lowest adsorption was found for Zn^2+^ for the two tested systems. The equilibrium in the examined systems was established after 56 h. After that time, 70.6% Pb^2+^, 56% Cu^2+^, 14.1% Zn^2+^ and 28.84% Cd^2+^ were removed from DW_PM, and 70.4% Pb^2+^, 84.5% Cu^2+^, 26.6% Zn^2+^ and 51.7% Cd^2+^ from SW_PM. The sorption affinity of the studied metals for the adsorbent changed in the following order: Pb^2+^ > Cu^2+^ > Cd^2+^ > Zn^2+^ in DW_PM, and Cu^2+^ > Pb^2+^ > Cd^2+^ > Zn^2+^ in SW_PM.

Kinetics of the metals binding by Fe_Alg beads in the presence of phosphate indicated that the pseudo-second-order model displayed the best correlation with adsorption kinetics data for all metals. The adjusted determination coefficients for the linear *t*/*a_t_* dependence as a function of time ranged from 0.982 to 0.999 (Table 2). Many studies published in the literature also report pseudo-second-order kinetics for adsorption of: Cd^2+^ and Cu^2+^ by alginate–based attapulgit foams [30], Cu^2+^ ions by glutaraldehyde crosslinked humic acid-immobilized sodium alginate [31], Cd^2+^, Cu^2+^, and Pb^2+^ ions by alginate modified with the grafting of urea and biuret [32] and Pb^2+^ ions by biocharealginate beads [33]. The values of *k*_2_ calculated for the models describing adsorption of all tested metals from the DW_PM solution were higher compared to *k*_2_ values calculated for the SW_PM solution. It indicates that adsorption of metals was faster in the DW_PM solution. Among the examined metals, the binding process was the fastest in the case of Cd^2+^, for which *k*_2_ was 0.422 ± 0.075 g/mg h in DW_PM and 0.059 ± 0.022 g/mg h in SW_PM. This process was the slowest in the case of Pb^2+^, for which *k*_2_ was 0.018 ± 0.005 g/mg h in DW_PM and 0.010 ± 0.003 g/mg h in SW_PM.

**Figure 4:**
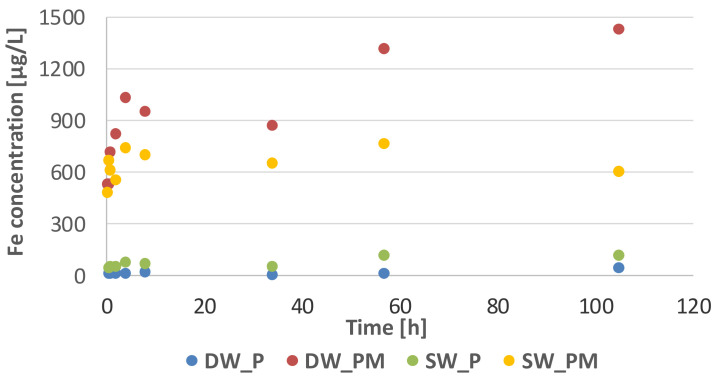
Changes in iron concentration in the tested solutions during the adsorption process. DW_P—deionized water enriched with phosphate, SW_P—effluent from a sludge enriched with phosphate, DW_PM—deionized water enriched with phosphate and metal ions, SW_PM—effluent from a sludge enriched with phosphate and metal ions.

**Figure 5:**
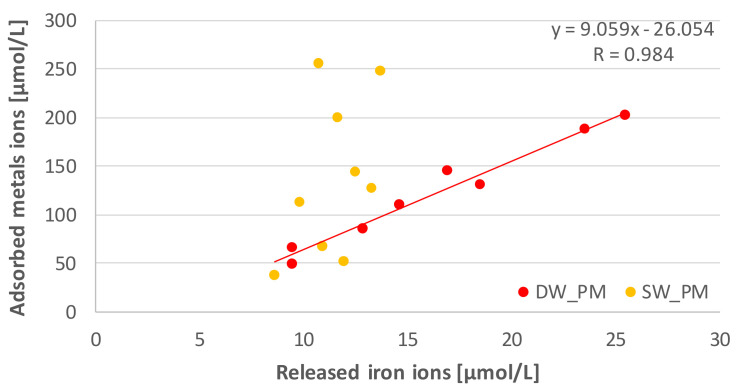
Relationship between the amount of iron released from alginate matrices cross-linked with Fe^3+^ ions and the total amount of metal ions adsorbed from a 1 L solution.

During the adsorption process, the release of iron ions from Fe_Alg beads took place. The most iron was released by systems enriched with metals (Figure 4). After completion of the tests, the DW_PM system released on average about 35 times more iron compared to the DW_P system, while the SW_PM system released on average about 5 times more iron than the SW_P system. Iron ions were released into the solution mainly during the first four hours of adsorption, as was the ion binding on the adsorbent. A positive correlation with a high correlation coefficient (R = 0.894) was calculated between the amount of released phosphate ions and the amount of adsorbed metal ions in the DW_PM solution, no such correlation was found in the SW_PM solution (Figure 5).

### 2.2. Phosphate Adsorption Isotherm

**Figure 6:**
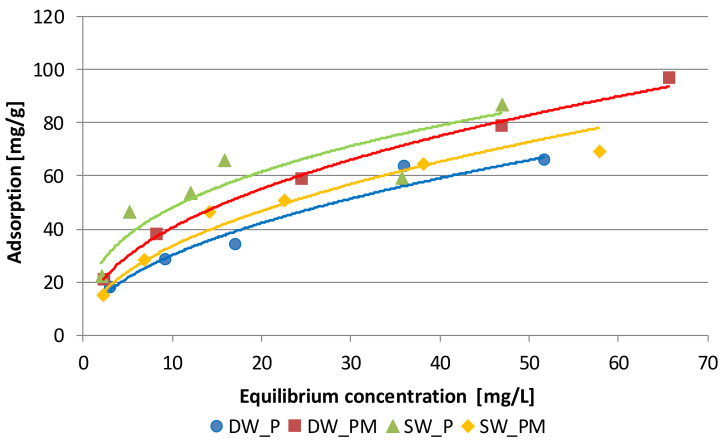
Freundlich isotherms of phosphate adsorption onto alginate matrices cross-linked with Fe^3+^ ions. DW_P—deionized water enriched with phosphate, SW_P—effluent from a sludge enriched with phosphate, DW_PM—deionized water enriched with phosphate and metal ions, SW_PM—effluent from a sludge enriched with phosphate and metal ions.

According to the Giles classification [34], the shape of the obtained phosphate adsorption isotherms onto Fe_Alg beads indicates that they are L-type isotherms of subgroup 1 (Figure 6). The isotherms are most commonly found in solute adsorption in aqueous solution and it was indicated that the adsorption occurs due to relatively weak forces, such as van der Waals forces and a low competition from solvent molecules [35]. The same form of isotherm was received by authors examining the process of phosphate adsorption on various adsorbents, e.g., goethite [12], wood fiber treated with carboxymethyl [18] and surfactant-modified natural zeolite [36].

This isotherm type is usually described by the Freundlich or Langmuir models. The phosphate adsorption system from DW_P and DW_PM was better described by the Freundlich model, because the value of *R_adj_*^2^ was higher than the value of this parameter calculated for the Langmuir model (Table 3). This type of isotherm presumes that the multilayer of the adsorption process occurs on a heterogenous surface. The values *k_F_* were higher in the isotherm describing adsorption in DW_PM, which indicates that in the equilibrium state, a higher sorption capacity of Fe_Alg beads relating to phosphate was in this system.

The phosphate adsorption from SW_P and SW_PM systems was better described by the Langmuir model: values of *R_adj_*^2^ were very high: 0.994 and 0.993, respectively. This model indicates that adsorption is proportional to the fraction of surface of the open adsorbent. The value *k_L_* was higher in the isotherm describing adsorption in the SW_P solution, which indicates that in the equilibrium state, a higher affinity of Fe_Alg beads relating to phosphate was in the SW_P system. In the presence of metals, this affinity decreased. Comparable values of *q_m_* in SW_P and SW_PM systems indicate a comparable number of active sites.

A comparison between the calculated parameters of adsorption isotherms and parameters calculated by other authors, who studied adsorption of phosphate ions on adsorbents containing iron, indicates comparable or higher sorption capacity of Fe_Alg beads relating to phosphate in the multicomponent solutions. For values of the *k*_F_ calculated for iron oxide tailing [15], goethite and alginate/goethite beads [37], Fe-Mn binary oxide [13] ranged from 11.7 to 27.2, while the calculated values of *q_m_* ranged from 8.2 to 108 mgPO_4_ g^−1^.

## 3. Discussion

Adsorption studies in multicomponent solutions, such as wastewater and their processing products, are important for the design, optimization and operation of purification technologies in the aquatic environment. In particular, heavy metal ion adsorption in multicomponent systems has been recognized as a strong antagonistic removal process, in which the properties and concentrations of compounds affect the adsorption efficiency [38,39,40]. The research conducted on the competitiveness of phosphate and multivalent metal adsorption by Fe_Alg showed that phosphate adsorption in the DW_PM solution was greater compared to the SW_PM solution. Intensification of phosphate adsorption in multicomponent solutions may be caused by multivalent metals forming additional active centers on the adsorbent. The mechanism of metal and phosphate adsorption on alginate gel matrices is different. Carboxylate function groups of alginate are negatively charged in neutral and alkaline media and hence, have greater affinity to cations. The sorption of polyvalent metal ions onto alginate takes place via a specific ion exchange mechanism involving the replacement of other cations by metal ions [41,42]. Studies on adsorption of multivalent metals to calcium-crosslinked alginate beads have shown that the metal uptake capacity at low pH is attributed to an ionic exchange protons [43,44] and showed high affinity for polyvalent metal ions, especially within a low-concentration region [45]. Alginate acid, in combination with multivalent metals, creates structures with large specific surface area and anionic sorption capacity, which enables phosphate binding on alginate adsorbents. Metals in the form of hydrogel capsules are characterized by higher sorption capacity per unit of metal, which is a consequence of the development of the adsorbent’s specific surface and large dispersion of metal cations on it [46], which can lead to the formation of more active sites on the adsorbent. Alginate is rich in carboxyl, hydroxyl and other active functional groups which can react with heavy metals through ion exchange or complex reaction [22,42]. The research carried out in this work showed that all the tested metals were adsorbed in solutions prepared in deionized water and in effluent water along with phosphate on Fe_Alg. During adsorption, partial exchange of the adsorbed metals with iron ions took place, which is indicated by a multiple increase in iron concentration in solutions containing DW_PM and SW_PM metals compared to DW_P and SW_P solutions. It suggests an ionic exchange process. This applies more to the DW_PM system, where a positive correlation was found between the amount of adsorbed metal ions and the amount of released iron ions. Studies on the relationship between the physical parameters of various metal ions, including toxic metal ions, and the binding affinity of these metal ions for alginate, have shown that Pb^2+^, Cd^2+^, Cu^2+^ and Zn^2+^ have higher affinity to alginates in comparison to Fe^3+^ [47,48]. In the DW_PM solution, the ionic exchange between metal ions and Fe^3+^ was four times greater than in the SW_PM solution, which is indicated by a much greater amount of released Fe^3+^ ions in the DW_PM solution. For the tested systems, the affinity of the Fe_Alg for metal ions was in the following decreasing order: Pb^2+^ > Cu^2+^ > Cd^2+^ > Zn^2+^ in DW_PM, and Cu^2+^ > Pb^2+^ > Cd^2+^ > Zn^2+^ in SW_PM. Metals’ adsorption in the DW_PM solution is consistent with other reports, in which the adsorption of these metals on calcium-crosslinked alginate adsorbents [49,50], on alginate modified by the grafting of urea maintained [51] and on activated carbon-containing alginate adsorbent [52] was studied. An affinity of Fe_Alg for number of metal ions found in the study are consistent with the ability of sodium alginate to bind to multivalent cations following the sequence of Pb^2+^ > Cu^2+^ > Cd^2+^ > Ba^2+^ > Sr^2+^ > Ca^2+^ > Co^2+^ > Ni^2+^ > Zn^2+^ > Mn^2+^ [53].

The second mechanism of metal binding in alginate is the formation of coordination complexes [42,53]. According to the metals’ classification of Nieboer and Richardson [54], lead belongs to type B metals and is characterized by a high value of the covalent index, i.e., strong ability to accept electrons from the ligand donor atom, and high value of the ion index, which is a measure of the possibility of ionic bond formation. Lead ions have the highest values of these two parameters among environmentally important metals. As a result, lead ions in a neutral environment and in the presence of various ligands with donor atoms form one of the most stable complexes. This explains why lead adsorption was high and comparable in the both of the tested systems: DW_PM and SW_PM. The other examined metals: copper, cadmium and zinc, belong to the intermediate-type metals and their ions are characterized by lower value of the covalent index (copper has the largest one) and the ion index compared to lead ions. Analysis via Fourier transform infrared spectroscopy (FTIR) of the sodium alginate crosslinking with CaCl_2_ after adsorption from tetra metallic mixture solution, Cu^2+^, Zn^2+^, Ni^2+^ and Cd^2+^, showed that metal ions bind to carboxyl and hydroxyl groups. The adsorption experiments demonstrated that the beads exhibited a high removal efficiency for the selective adsorption of Cu^2+^. It was due to better bond stability with Cu^2+^ compared to other metals [50]. Research conducted by Chen et al. [42] showed that the nature of lead uptake was the typical ion exchange between Ca^2+^ and Pb^2+^ at the carboxylate anionic site, whereas the copper uptake took place through the ion exchange between Ca^2+^ and Cu^2+^ as well as the formation of the coordination complex. These different binding mechanisms may cause a greater adsorption of copper than that of lead and a relatively low adsorption of phosphate in the SW_PM solution. The higher adsorption of metals, found in the SW_PM (pH 6.8) solution compared to DW_PM (pH 5.6), could have been influenced by the small pH difference between these solutions. Numerous experimental results indicate that pH is one of the most crucial factors influencing the efficiency of the metal ion uptake process. It is justifiable, as it is able to change both the surface properties of the bio-sorbent and the metal ion form in the bulk solution. The former factor is usually modeled by assuming the effect of competition between the metal ions and protons for the available binding sites or by applying the ion-exchange models, in which binding of the metal ion occurs in the reaction with protonated surface sites, accompanied by release of proton(s) [49]. Studies of ion exchange of various metals in alginate under different pH conditions showed that this exchange significantly decreased with decreasing pH [48]. The exception in the experiment was lead, the adsorption of which in the DW_PM and SW_PM solution was at a comparable level. Greater adsorption of the investigated metals of the intermediate type in the SW_PM solution may result from the presence of various types of compounds that, after adsorption, can form complex compounds with these metals. Intermediate-type metals, compared to B-type metals, have stronger tendency to complex with ligands other than water, and form complexes with donor atoms, such as oxygen, nitrogen and sulfur [54].

The research carried out showed that in the solution prepared based on distilled water, the addition of metals increased the adsorption of phosphate, which is indicated by the higher value of adsorption at equilibrium state in the DW_PM solution compared to DW_P, and the higher value of *k_F_* in the Freundlich isotherm calculated for the phosphate adsorption process in these systems. The increase in phosphate adsorption in a solution containing only phosphate and metals compared to a solution containing only phosphate indicates a slight synergistic effect of metals on phosphate adsorption. This process was best described by the Freundlich model, which is characteristic of multilayer adsorption. The slower adsorption rate of phosphate in the DW_PM solution compared to the SW_PM solution could be related to the formation of subsequent layers. In the SW_PM metal-enriched effluent water, the adsorption of phosphate on Fe_Alg was lower compared to the adsorption in the SW_P system. The addition of metals reduced phosphate adsorption, which is indicated by the lower value of adsorption at equilibrium state in the SW_PM solution compared to SW_P, and the *k_L_* parameter in the Langmuir isotherm calculated for the phosphate adsorption process in this system. This process was best described by the Langmuir model, which is characteristic of monolayer coverage and no later interaction between adsorbed molecules. Calculated reaction rate constants in the pseudo-second-order equation show that the process was much faster than for solutions containing only phosphate and metals (DW_P and DW_PM). This could be due to the formation of a single layer of adsorbed components, largely formed by metals, as indicated by their greater adsorption in the SW_PM solution compared to DW_PM. Results indicated that phosphate and multivalent metal ions in effluent water might show competitive adsorption. Less phosphate adsorption in this system could also be caused by the presence of compounds in effluent water that, in the presence of metals, compete with phosphate for active sites on the adsorbent surface. Metal ions studied at work can form complexes with donor atoms, such as oxygen, nitrogen and sulfur. These complexes could hinder the adsorption of phosphates in the SW_PM solution. As a result of the adsorption of Cu^2+^, Zn^2+^ and Cd^2+^, new active sites could be created, which had a greater affinity for other SW compounds of anionic nature then phosphate. Such anion behavior was observed during the study of the effect of the coexisting ions on the adsorption of fluorides on the alginate gelled with zirconium in wastewater. The presence of HCO_3_^−^, SO_4_^2−^ and PO_4_^3−^ had a large negative impact on fluoride removal. The decreased defluorination was attributed to the lower affinity of zirconium alginate for fluoride and a competition between the fluoride ions and the other anions [55].

The conducted research indicates that the possibility of using phosphate ion adsorption on alginate matrices to recover phosphorus from liquid waste in sewage treatment plants is limited. To limit the competitive effect of metals on the phosphate adsorption process, the method should be used in municipal wastewater treatment plants in small, non-industrial agglomerations. Treated effluent water must not contain toxic metals, such as lead and cadmium, which do not perform any biological functions. In the case of other metals, tests should be carried out to determine their limit concentrations, which do not pose a threat to the environment.

## 4. Materials and Methods

### 4.1. Materials

Alginate matrices, cross-linked with Fe^3+^ ions (Fe_Alg) with bead size 2.5–3.0 mm, were used as adsorbents. The hydrogel beads were produced with the injection method. Nine grams of sodium alginate powder (Keltone HV, ISP-Germany, Marl, Germany) was dispersed in 600 mL of deionized water to give a 1.5% *w*/*v* alginate solution. This solution was mixed with a mechanic stirrer until a transparent, viscous solution was obtained. In order to receive a solution containing fully hydrated polymer chains, the mixing process was carried out with use of a magnetic agitator for 24 h. Hydrogel capsules of alginate/Fe(III) were received by dosing the sodium alginate in a quantity of 5 mL each time with the classic 10 mL syringe (Polfa S.A., Lublin, Poland), ended up with a needle diameter 0.8 × 40 mm (TERUMO, Belgium, Levuen, Belgium), to 100 mL FeCl_3_ 0.155 M solution, at 2–3-min intervals. Time of the reaction after injection of the polysaccharide solution was 70 min. During forming the beads, the FeCl_3_ solution was stirred continuously at 600 rpm. The beads were rinsed with distilled water, the liquid was drained, and the adsorbent was sealed in a deionized water (DW) container.

The following salts were used to prepare the adsorbed ion solutions:

CuSO_4_, (CH_3_COOH)_2_Cd, Pb(NO_3_)_2_, ZnSO_4_ and KH_2_PO_4_ (EUROCHEM BGD, Tarnów, Poland). The solutions were prepared in DW and by enriching water effluent from a sludge thickener press (SW) with them, some selected qualitative indicators of SW are shown in Table 4. The SW solution comes from a mechanical-biological sewage treatment plant (Nowogard in Poland) with a capacity of 3400 m^3^/d.

### 4.2. Analytical Methods

Determinations of tested water quality indicators were carried out using standard methods [56]. Contents of total metals (Cu, Cd, Pb, Zn and Fe) in the solutions were determined by means of Atomic Absorption Spectroscopy using a spectrometer ThermoElemental, Solaar S, Walthman, USA with atomization occurring in acetylene/air flame. Phosphate concentration was measured by the molybdenum blue colorimetric method [57] using a two-beam spectrophotometer Techcomp UV/VIS 8500 at 890 nm wavelength.

### 4.3. Adsorption Kinetic Measurements

The phosphate adsorption kinetics on Fe_Alg beads were studied in 10 mg PO_4_ L^−1^ solutions prepared on the basis of DW and SW without addition of DW_P and SW_P, as well as with the addition of metal ions: Cu^2+^, Cd^2+^, Pb^2+^ and Zn^2+^ (DW_PM and SW_PM) at the concentration of each metal ion of 10 mg/dm^3^ and a constant ionic strength of 30 mmol against KCl, as proposed by Naira et al. [58]. A volume of 50 mL of prepared solutions and 0.01 g dry matter Fe_Alg matrices was added into the conical flasks with a capacity of 100 mL. Nine measuring series were prepared for each type of solution in duplicate. Changes in phosphate and metal contents (including iron) in the aqueous phase were tested in successive series after 0.25, 0.5, 1, 12, 4, 8, 34, 56 and 105 h. In parallel, the concentration of phosphate and metals in control samples without adsorbent was tested.

### 4.4. Phosphate Adsorption Experiments

Phosphate adsorption characteristics were studied in a static batch system proposed by Naira et al. [58]. The tests were carried out at 20 °C in DW and SW, which were enriched with phosphate(V) ions at a concentration of 10, 20, 30, 40, 60 and 80 mg L^−1^ (DW_P and SW_P), and metal ions: Cu^2+^, Cd^2+^, Pb^2+^ and Zn^2+^ at a concentration of 5 mg L^−1^ each (DW_PM and SW_PM), with a constant ionic strength of 30 mmol against KCl. A volume of 50 mL of prepared solutions and 0.01 g DS Fe_Alg matrices was added into the conical flasks with a capacity of 100 cm^3^. Changes in the content of phosphate ions and metals in the solution were tested at equilibrium after 56 h. The mixtures were shaken on a laboratory shaker for two hours in the beginning and the end of the adsorption process. The experiment was carried out in triplicate.

### 4.5. Data Analysis

The linearized form of equations was used to determine the parameters of mathematical models describing the kinetics of the adsorption process studied. Kinetic data of phosphate sorption on Fe_Alg was described using the pseudo-second-order rate equation developed by Ho and McKay [59,60] (Equation (1)):

(1) $$\frac{da_{t}}{dt}=k_{2}\cdot {\left(a_{eq}-a_{t}\right)}^{2}$$

The solution to this equation under boundary conditions *t* = 0 to *t* = *t* and from *a_t_* = 0 to *a_t_* = *a_eq_* is Equation (2):

(2) $$\frac{1}{a_{t}}=\frac{1}{k_{2}a_{eq}^{2}}+\frac{t}{a_{eq}}$$

where *k*_2_ is the second-order rate constant of adsorption, and *a_eq_* is the amount of phosphate adsorbed at equilibrium [61].

The equilibrium data for the removal of phosphate in the present investigation were analyzed using the two-parameter model, Langmuir (Equation (3)) and Freundlich (Equation (4)) isotherms:

(3) $$q_{e}=\frac{q_{m}k_{L}C_{e}}{1+k_{L}C_{e}}$$

(4) $$q_{e}=k_{F}C_{e}^{1/n_{F}}$$

where *C_e_* is the equilibrium concentration of phosphate in the solution, *q_e_* is the correspondent uptake capacity of the adsorbent, *q_m_* is the maximum adsorption capacity, *k*_L_ is the affinity constant (Langmuir constant), *k*_F_ and *n*_F_ are Freundlich constants and 1/*n_F_* is the heterogeneity factor.

The linear equations of Langmuir and Freundlich are represented as follows (Equations (5) and (6), respectively).

(5) $$\frac{C_{e}}{q_{e}}=\frac{1}{k_{L}\left(q_{m}\right)}+\frac{C_{e}}{q_{m}}$$

(6) $$logq_{e}=\log k_{F}\;+\;\frac{\left(logC_{e}\right)}{n_{F}}$$

Linear regression analysis has been used to determine the best-fit isotherm and the method of least squares has been used for finding parameters of the equations. For the fitting-degree of the isotherms and kinetic equation with the experimental data, the adjusted coefficient of determination, *R_adj_*^2^, was used. The *R_adj_*^2^ is a good tool in selecting models, which takes into account the experimental degrees of freedom (*n*1), where *n* is the number of data points [35]. All statistical analyses were performed with the software STATISTICA version 13.3.

## Figures and Tables

**Table 1:** Estimated pseudo-second-order kinetic model parameters for phosphate adsorption by alginate matrices cross-linked with Fe^3+^ ions. DW_P—deionized water enriched with phosphate, SW_P—effluent from a sludge enriched with phosphate. DW_PM—deionized water enriched with phosphate and metal ions, SW_PM—effluent from a sludge enriched with phosphate and metal ions.

Solution	Adsorption at Equilibrium State	Rate Constant of Adsorption	Initial Adsorption Rate	Adjusted Coefficient of Determination
	*a_e__q_* (mgPO_4_/g)	*k*_2_ (g/mg h)	*V_o_* (mg/g h)	*R_adj_* ^2^
DW_P	18.83 ± 0.32	0.011 ± 0.002	4.17 ± 0.85	0.998
DW_PM	22.27 ± 0.51	0.028 ± 0.011	13.79 ± 5.28	0.996
SW_P	22.83 ± 0.12	0.047 ± 0.015	24.39 ± 7.79	0.999
SW_PM	15.11 ± 0.12	0.067 ± 0.022	15.41 ± 5.12	0.999

**Table 2:** Estimated pseudo-second-order kinetic model parameters for metals adsorption by alginate matrices cross-linked with Fe^3+^ ions. DW_PM—deionized water enriched with phosphate and metal ions, SW_PM—effluent from a sludge enriched with phosphate and metal ions.

System	Metal	Adsorption at Equilibrium State	Rate Constant of Adsorption	Initial Adsorption Rate	Adjusted Coefficient of Determination
		*a_eq_* (mgPO_4_/g)	*k*_2_ (g/mg h)	*V_o_* (mg/g h)	*R_adj_* ^2^
DW_PM	Pb	19.12 ± 0.39	0.018 ± 0.005	6.49 ± 1.89	0.997
	Zn	4.13 ± 0.03	0.422 ± 0.075	7.18 ± 1.28	0.999
	Cd	7.50 ± 0.03	0.166 ± 0.035	9.35 ±1.98	0.997
	Cu	15.08 ± 0.12	0.059 ± 0.011	13.48 ± 2.50	0.998
SW_PM	Pb	19.42 ± 0.56	0.011 ± 0.003	4.06 ± 1.02	0.993
	Zn	7.23 ± 0.16	0.059 ± 0.022	3.10 ± 1.15	0.999
	Cd	12.52 ± 0.19	0.042 ± 0.010	6.58 ± 1.59	0.997
	Cu	22.94 ± 1.03	0.010 ± 0.003	5.07 ± 1.63	0.982

**Table 3:** Freundlich and Langmuir isotherm constants for phosphate adsorption onto alginate matrices cross-linked with Fe^3+^ ions. DW_P—deionized water enriched with phosphate, SW_P—effluent from a sludge enriched with phosphate, DW_PM—deionized water enriched with phosphate and metal ions, SW_PM—effluent from a sludge enriched with phosphate and metal ions.

Solution	Freundlich Model *			Langmuir Model **		
	1/*n*_F_	*k*_F_	*R_adj_* ^2^	*q_m_* (mgPO_4_ g^−1^)	*k*_L_ (L mgPO_4_^−1^)	*R_adj_* ^2^
DW_P	0.49 ± 0.06	9.98 ± 0.89	0.968	89.3 ± 21.9	0.055 ± 0.008	0.913
DW_PM	0.44 ± 0.01	14.5 ± 0.34	0.998	112 ± 32.0	0.063 ± 0.007	0.947
SW_P	0.42 ± 0.08	19.8 ± 3.26	0.898	100 ± 11.8	0.135 ± 0.005	0.994
SW_PM	0.48 ± 0.05	11.0 ± 0.95	0.964	83.5 ± 8.01	0.083 ± 0.003	0.993

* Freundlich $q_{e}=k_{F}C_{e}^{1/n_{F}}$; ** Langmuir $q_{e}=\frac{q_{m}k_{L}C_{e}}{1+k_{L}C_{e}}$

**Table 4:** General characteristics of the tested effluent from a sludge.

Water Quality Indicators	Value	Water Quality Indicators	Value
pH	7	N_Ammonia, mg/L	12.19
RedOx Potential Eh, mV	−36.6	N_Nitrite, mg/L	0.03
Color, mgPt/L	19 s	N_Nitrate, mg/L	7.43
turbidity, (Nephelometric Turbidity Units) NTU	65.8	Cu^2+^, mg/L	0.0065
Hardness, CaCO_3_ mg/L	220.4	Pb^2+^, mg/L	0.0341
Alkalinity, mval/L	4.1	Zn^2+^, mg/L	0.0592
Phosphate, mg/L	8.06	Cd^2+^, mg/L	0.0025
